# Effects of body awareness therapy on balance and fear of falling in patients with chronic obstructive pulmonary disease: a randomized controlled trial

**DOI:** 10.1186/s13030-024-00303-x

**Published:** 2024-02-26

**Authors:** seda karaca, Aysel Yildiz Özer, Sait Karakurt, Mine Gülden Polat

**Affiliations:** 1 Department of Physiotherapy and Rehabilitation, Cardiopulmonary Rehabilitation Department, Faculty of Health Sciences, Marmara University, Süreyyapaşa Başıbüyük Street, Number:4, B, Maltepe, Istanbul, 34854 Turkey; 2 Department of Pulmonary Medicine, School of Medicine, Marmara University, Istanbul, Turkey

**Keywords:** Body awareness therapy, Copd, Balance, Fear of falling

## Abstract

**Background:**

Assessment of extrapulmonary comorbidities is essential in chronic obstructive pulmonary disease (COPD). Deterioration of balance and increasing fear of falling are two of the most significant extrapulmonary manifestations. Although pulmonary rehabilitation (PR) is well-known and effective for COPD patients, there is a need for alternative treatments to enhance balance and alleviate concerns about falling. This study aimed to investigate the effect of Body Awareness Therapy (BAT), in addition to the PR program, on balance and fear of falling in patients with COPD.

**Methods:**

Forty-three patients were randomized into two groups: the BAT + PR group (BAT: once a week, 60 min + PR: 30 min, seven days of the week) or the PR group (PR: 30 min, seven days of the week) for eight weeks. Primary (balance, fear of falling) and secondary (dyspnea, muscle strength, functional capacity) outcomes were assessed at two different times: the baseline and end of the eight weeks.

**Results:**

Significant improvements were found in dynamic balance (reaction time η^2^ = 0.777, movement velocity η^2^ = 0.789, endpoint excursion η^2^ = 0.687, maximal excursion η^2^ = 0.887), static balance on firm ground (eyes opened η^2^ = 0.679, eyes closed η^2^ = 0.705), dyspnea (η^2^ = 0.546), muscle strength (η^2^ = 0.803), and functional capacity (η^2^ = 0.859) of the BAT + PR group (*p* < 0.05 for all). The improvement in fear of falling was significantly greater in the BAT + PR group than in the PR group (*p* < 0.001, η^2^ = 0.331).

**Conclusion:**

The BAT method added to PR was more effective than PR alone in improving balance and reducing the fear of falling in COPD patients.

**Trial registration:**

This randomized controlled study was registered at clinicaltrials.gov, NCT04212676, Registered 28 December 2019.

**Supplementary Information:**

The online version contains supplementary material available at 10.1186/s13030-024-00303-x.

## Background

Chronic Obstructive Pulmonary Disease (COPD) is an avoidable and manageable respiratory disease that causes ongoing breathing difficulties and limitations in the airways and/or air sacs of the lungs, often due to prolonged exposure to harmful gases or particles [1, 2]. COPD treatment aims to manage the disease, prevent disease progression, and exercise tolerance [2]. The GOLD (Global Initiative for Obstructive Lung Diseases) and other treatment guidelines for chronic lung diseases emphasize that COPD is a systemic disease accompanied by extrapulmonary involvement. These factors adversely affect both the severity of the disease and the prognosis [1–3]. The most common extrapulmonary comorbidities in COPD are muscle weakness, balance disorders, and an increased risk of falls. [4, 5]. Peripheral muscle weakness, systemic inflammation, physical inactivity, and decreased exercise capacity affect balance in patients with COPD [6–8]. Clinical studies have shown that patients with COPD have limitations in functional capacity and balance compared to healthy individuals [7, 9, 10]. It has been reported that patients with COPD often experience functional performance deficits that affect their postural stability and balance due to lower extremity muscle weakness, an inability to manage symptoms, a worsening ability to adapt to the disease, and reduced physical activity levels [7, 9, 11]. In a study by Beauchamp et al., it was found that COPD patients had decreased responses to postural perturbations compared to healthy individuals [11]. In a comprehensive study characterizing proprioceptive postural control in COPD, it was found that patients used more ankle muscle proprioceptive signals and less back muscle proprioceptive signals during balance control [12]. Furthermore, achieving successful balance depends not only on motor performance but also on mental awareness, the individual's ability to harmonize with the environment and space, and the effective use of proprioceptive inputs [8, 11, 12]. Increased loss of balance may constitute a potential risk factor for the possibility of falls in patients with COPD [13]. Lower extremity muscle weakness and loss of balance ability are the two main factors that cause falls in COPD patients [13, 14].

Pulmonary rehabilitation (PR) provides minimal gains for extrapulmonary comorbidities such as balance in COPD [15, 16]. Therefore, alternative treatment modalities are needed to increase balance skills and prevent falls. Body Awareness Therapy (BAT) is an alternative method that treats patients holistically and attaches importance to the quality, coordination, and rhythm of movement [17, 18]. Jacques Dropsy, a French exercise instructor and psychotherapist, developed BAT as an alternative method in the early 1970s [19]. It was later adapted to rehabilitation programs by Swedish and Norwegian physiotherapists. The treatment includes proprioceptive exercises that aim to improve movement quality, rhythm, and coordination by focusing on the senses [17]. This holistic approach addresses the physical, psychological, and social aspects of the individual. The treatment program is tailored to the patient's needs and focuses on the body's movements [19, 20]. It includes exercises that enhance body awareness, carefulness, and sense of position. BAT builds upon traditional physiotherapy methods [21]. Breathing exercises, relaxation exercises, sensory awareness development, positioning, body stabilization, rhythm and coordination development, and techniques related to the ability to perform quality exercise with maximum awareness are among the physiotherapy methods used in BAT [21, 22]. Upon reviewing the literature, it is evident that BAT can alleviate pain, fatigue, eating, and sleep issues in various patient groups [17, 19, 20]. Additionally, it can improve exercise quality, coordination, balance, postural control, quality of life, and mind–body integration. BAT has become increasingly popular among health professionals, particularly physiotherapists, in recent years. [19, 22, 23]. It has been widely used and has been shown to have many benefits in the treatment of various medical conditions such as chronic pain, fibromyalgia, osteoarthritis, eating disorders, sexual traumas, coronary artery diseases, congestive heart failure, falls in old age, anxiety, and depression [18–23].

COPD is a multisystemic disease that can lead to musculoskeletal, cardiac, metabolic, and psychological comorbidities [1]. Comorbid conditions can have a negative impact on the severity of the disease and the prognosis [1, 3]. Recent studies have shown that loss of balance is one of the comorbid conditions in these patients [9, 11, 15]. The incidence of falls is higher in COPD patients, with a 55% increase compared to non-COPD patients [14]. Fear of falling can lead to limitations in daily life, self-care activities, social isolation, introversion, and depression, which can seriously deteriorate the quality of life and negatively affect the disease prognosis [14, 24].

In clinic BAT applications, maintaining postural stability, relaxed breathing, and awareness are crucial for achieving dynamic balance and reducing fear of movement. The exercises and training included in the program focus on how individuals manage their balance and relate to the ground and vertical axis in their environment [21, 22]. Although BAT comprises simple, repetitive movements that preserve the ability to use stability limits, one of its main components is to enable the participant to experience their body and its limits through multiple slow, repetitive movements. This is made possible thanks to the neuroplasticity of the brain. Movement awareness develops through the cortical processing of movement-related information in the brain and the facilitation of proprioceptives [17]. It can improve postural control, balance, and coordination while reducing fear of movement [19, 22]. Increasing awareness of body responses, movement limits, and environmental inputs may reduce the fear of falling in individuals who are confident in their balance ability.

BAT is a method that aims to normalize posture, balance, muscular tension, and stiffness in many conditions. It is a principled movement training technique that emphasizes sensory-motor awareness and movement teaching based on cognitive perception of movement [17, 18]. It can liberate the individual both psychologically and physically by providing awareness. It is anticipated that patients will improve their mind–body-behavior interaction through learned experiences [19]. This may contribute to less energy consumption, higher participation and performance in daily life, and the ability to manage dyspnea, fear of movement, and postural control.

With these features, BAT can improve the ability of patients with COPD to perform their daily living activities with less energy consumption and develop their ability to cope with their disease. This study aimed to investigate the effect of BAT on balance and fear of falling in patients with COPD.

## Methods

### Participants

The study included 43 patients who met the inclusion criteria of patients diagnosed with COPD by the pulmonologist at Marmara University Faculty of Medicine and referred to the PR Laboratory of the Faculty of Health Sciences between October 2020 and April 2023. The inclusion criteria were as follows: being diagnosed with COPD according to Global Initiative for Chronic Obstructive Lung Disease (GOLD), being in GOLD stage 1, 2, or 3, volunteering to participate in the study, being between the ages of 40–65, being in a clinically stable period, not having serious hearing and vision problems and not showing signs of exacerbation within two months. The exclusion criteria were as follows: having diagnosed vision, hearing, vestibular, cognitive, or neurological problems that may affect balance, diagnosed orthopedic/rheumatic problems affecting mobility or a history of surgery, presence of cardiac problems that prevent participation in exercise and/or unstable cardiac conditions, and receiving pulmonary rehabilitation within 12 months.

### Randomization

**Fig. 1:**
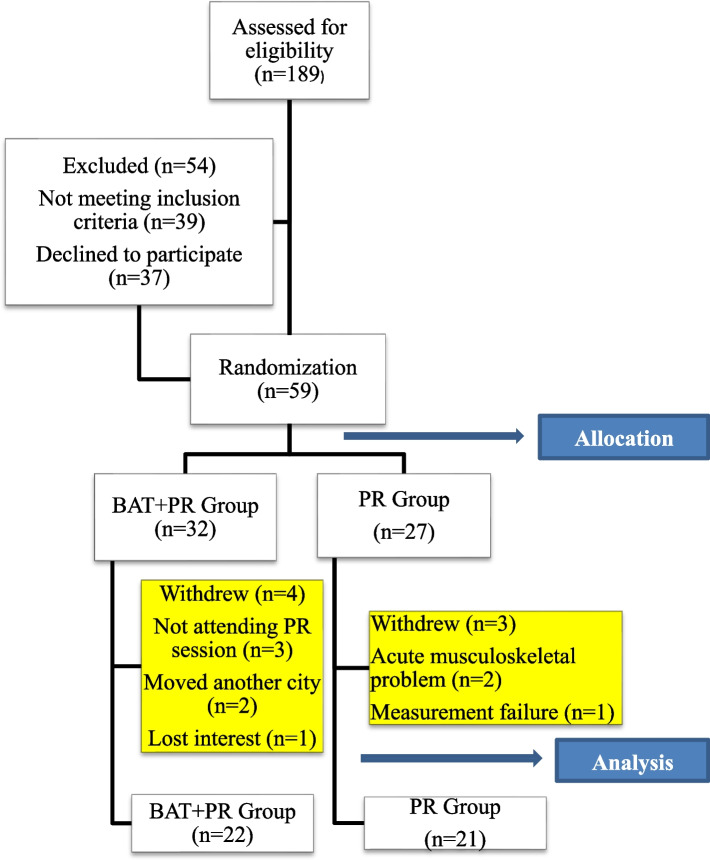
The study flow chart

A computer-based randomization program [25] was used to determine the patients to be assigned to the experimental and control groups. Two number sequences consisting of 256 numbers were determined by random selection from numbers 1 to 256; the first of these number sequences was accepted as the Body Awareness Therapy + Pulmonary Rehabilitation Group (BAT + PR) and the second as the Pulmonary Rehabilitation Group (PR). Figure 1 shows the study flow chart.

### Intervention

All patients in both groups were given a 30-min PR program every day for eight weeks. The physiotherapist who conducted the BAT program in this project completed her BAT education four years ago and wrote her master's thesis on this subject. The researcher is still conducting patient training in the clinic. An 8-week BAT program was conducted for this study. Exercises were planned individually and progressed gradually. Exercises were integrated with diaphragmatic breathing and tailored to their specific needs. As part of the program, patients were given homework to record their feelings during different environmental conditions, activities, and exercises. Participants were requested to evaluate their stress, fear, and anxiety levels during daily activities and relaxation exercises. The individual experiences of participants were discussed via weekly interviews. The physiotherapist supervised three PR sessions each week, while the remaining sessions were conducted at home and monitored via phone. At the end of eight weeks, the initial evaluations were repeated.

PR Group: This group participated in an 8-week PR program. The PR program consisted of diaphragmatic breathing exercises, thoracic expansion exercises, cough improvement practices, lower and upper extremity strength and endurance exercise training 3 times a week. Participants were given activity suggestions, self-management education and taught energy conservation techniques [26].

BAT + PR Group: This group participated in BAT in addition to PR. BAT was performed once a week for 8 weeks. The BAT program consisted of specific exercises focusing on how the body is used during movements, aiming to improve the patient's awareness of the body and ability to be mindful [17, 18]. Among the physiotherapy methods used in BAT, most are breathing exercises, relaxation exercises, body scanning, movement approaches specific to sensory awareness, positioning, stabilization, improving rhythm and coordination, and improving the ability to perform exercises with maximum awareness [18, 20, 27]. The activities included relaxation exercises, breathing exercises, floor exercises for trunk and pelvic movements, weight-bearing using the extremities, and proprioceptive movements. Movements were performed in various positions to determine the body's centerline. All exercises were started in the supine position to maximize proprioceptive movement feedback and minimize energy requirements during tasks [20]. Movements were taught in a series of lessons over eight weeks, with preprogression of movements selected based on the participant’s comfort (Supplement 1).

### Primary and secondary outcomes measures

The primary outcomes were to assess the difference in balance and fear of falling parameters between groups. The levels of static and dynamic balance of the patients were evaluated with the Balance Master System balance and performance test device (NeuroCom System Version 8.1.0, NeuroCom® International Inc. USA), which is an objective measurement tool [28]. Modified Clinical Test of Sensory (mCTS) for Static Balance: Positional changes were detected while the patient was on a force platform and on one leg in a soft setup, with eyes open and closed. The force platform of the system is sensitive to the patient’s center of gravity. Each test took 10 s, and was repeated three times, and the average oscillation was calculated. Finally, mean values were taken for both floors, and composite scores were calculated.

Limits of Stability Test (LOS) for Dynamic Balance: The patients were asked to reach eight different points on the computer screen in the fastest and most linear way by shifting the center of gravity while standing still on the center point determined on the platform. The LOS test measured several parameters, including reaction time (RT), movement velocity (MVL), endpoint excursion (EPE), maximal excursion (ME), and directional control (DC). RT is defined as the time in seconds between the signal to move and the initiation of movement. MVL is the average speed of center of gravity (COG) movement, expressed in degrees per second, between 5 and 95% of the distance to the primary endpoint. Endpoint excursion (EPE) is defined as the distance traveled by the COG on the primary attempt to reach the target. Maximal excursion (ME) is the furthest distance traveled by the COG during the trial, which may exceed the endpoint excursion if the subject makes additional corrective attempts. In addition to the composite score, the LOS scores from the eight transitions were combined to provide an average score for each of the four main directions: forward, backward, right, and left.

Fear of falling evaluated with the Turkish version of the Fall Efficiency Scale International (FES-I) is a 10-item scale that includes bathing, lying on shelves, walking around the house, preparing meals, getting into and out of bed, looking at the door, sitting and getting up from a chair, and getting dressed [29].

The secondary outcomes were to assess the difference in functional capacity, muscle strength, and dyspnea parameters between groups. The Modified Borg Dyspnea Scale (BDS), based on scoring dyspnea severity between 0 and 10, was used after the 6 Minute Walking Test (6MWT). A MicroFET® 2 (Hoggan Scientific; USA) electronic hand dynamometer was used to measure quadriceps muscle strength in the dominant extremity. Functional capacity was assessed with the 6MWT.

### Statistical analysis

In this study, data analysis was performed by a blinded expert. Normality distributions were evaluated with histogram curves and the Shapiro–Wilk test. The scores of the normally distributed variables were expressed with mean and standard deviation values. The scores of the normally distributed variables were expressed with mean and standard deviation values. Independent Sample T test was used to determine the differences between the groups. The difference in dichotomous variables was analyzed with the chi-square test. Variance analyses were performed using ANOVA to determine whether the protocols affected the primary and secondary outcomes. While *p* < 0.05 was used for statistical significance, partial eta squared (η^2^) was used to interpret the clinical significance of the results. The interpretation of eta squared values is based on the following references: η2 = 0.01 indicates a small effect, η2 = 0.06 indicates a medium effect, and η2 = 0.14 indicates a large effect [30].

G Power 3.1.9.7 software was used to calculate the sample size and power of the study. A study in which BAT was performed in the COPD population or chronic respiratory diseases in common with primary outcome measures to determine sample size could not be found. For this reason, the study that investigated assessing balance in COPD patients in the same GOLD stage group as the patients in this study was selected. The Berg Balance Test results in the study of Beauchamp et al. were used as a reference to calculate a priori power analysis [31]. Accordingly, when the effect size was entered as 1.00, the alpha error as 5%, the power as 80%, and the ratio of the number of people in the groups as 1:1, it was calculated that the minimum required sample size should be 34 people. Considering the possibility of data loss in the study, 30% more individuals than the minimum required sample size were included in the evaluation. When the post-hoc power was calculated after the end of the study, the equilibrium parameter, which is the primary output of this study, was taken as a reference. When the effect size of the reference parameter based on the difference between the groups was 1.99, the alpha error was 0.05, and the hypothesis was selected as two tails, the power of the study was calculated as 89%.

## Results

Forty-three patients completed the study. According to the baseline values, while 45.55% of the patients in the BAT + PR group participating in the study were female, this ratio was 38.09% in the PR group (*p* = 0.625). The mean age of the BAT + PR group was 52.59 ± 7.03 years, and that of the PR group was 53.38 ± 7.17 years (*p* = 0.717). The severity of the disease examined by the CAT score was found to be 23.95 ± 9.96 in the BAT + PR group. The CAT score of the PR group was found to be 25.14 ± 9.03 (*p* = 0.685). Information on other demographic characteristics is shown in Table 1.

**Table 1:** Demographic characteristics of patients

Variables		Groups		P
		**BAT + PR**	**PR**	
**Gender**	**Female n (%)**	10 (45.45)	8 (38.09)	0.625^a^
	**Male n (%)**	12 (54.55)	13 (61.91)	
**Age (year)**		52.59 ± 7.03	53.38 ± 7.17	0.717^b^
**Smoke User (yes/no)**		13/9	11/10	0.773
**GOLD Stage (1/2/3)**		3/17/2	5/14/2	0,634
**CAT**		23.95 ± 9.96	25.14 ± 9.03	0.685^b^
**mMRC**		2.40 ± 1.40	2.09 ± 1.33	0.457^b^

^a^Pearson Chi-Square

^b^Independent T-test; *CAT* COPD Assessment Test, *PR* Pulmonary Rehabilitation Group, *FEV1/FVC* Forced Expiratory Volume during first second/Forced Vital Capacity, *BAT* + *PR* Body Awareness Therapy + Pulmonary Rehabilitation Group, *mMRC* Modified Medical Research Council

The primary outcomes of the study are Limits of the Stability (LOS), Modified Clinical Test of Sensory (mCTS), and Fear of Fall (FoF). The groups had no significant difference in any parameter according to the baseline values except muscle strength (*p* = 0.036) (Table 2).

**Table 2:** Comparison of baseline values of groups

Variables		Groups		P
		**BAT + PR**	**PR**	
**LOS**	**RT**	0.94 ± 0.07	0.93 ± 0.06	0.742
	**MVL**	3.40 ± 0.51	3.63 ± 0.67	0.216
	**EPE**	71.63 ± 7.27	72.19 ± 6.51	0.794
	**DC**	73.86 ± 5.59	72.80 ± 5.30	0.530
	**ME**	86.18 ± 8.65	86.23 ± 9.66	0.984
**mCTS**	**Firm EO**	0.76 ± 0.17	0.69 ± 0.23	0.290
	**Firm EC**	0.92 ± 0.18	0.88 ± 0.21	0.468
	**Foam EO**	1.12 ± 0.21	1.02 ± 0.19	0.114
	**Foam EC**	2.36 ± 0.13	2.31 ± 0.21	0.406
**FoF**		67.04 ± 20.83	64.00 ± 21.65	0.641
**Muscle Strength**		17.35 ± 2.01	18.50 ± 1.36	0.036*
**6-MWT**		426.95 ± 36.89	417.47 ± 23.13	0.321
**BDS**		4.04 ± 0.89	4.19 ± 1.03	0.625

Independent Sample T-test, *PR* Pulmonary Rehabilitation Group, *DC* Directional Control, *EC* Eyes Closed, *EO* Eyes Open, *EPE* End-point Excursion, *FoF* Fear of Falling, *BAT* + *PR* Body Awareness Therapy + Pulmonary Rehabilitation Group, *LOS* Limit of Stability, *mCTS* Modified Clinical Test of Sensory, *ME* Maximum Excursion, *MVL* Movement Velocity, *RT* Reaction Time, *6-MWT* 6-Minutes Walking Test, *BDS* Borg Dyspnea Scale

When the post-treatment values were compared, improvements were observed in all subscales of LOS except Directional Control (DC) (*p* = 0.956) in the BAT + PR group. There was a significant interaction effect for the groups by time in Reaction Time (RT) (η^2^ = 0.777, large), Movement Velocity (MVL) (η^2^ = 0.789, large), Endpoint Excursion (EPE) (η^2^ = 0.687, large), and Maximal Excursion (ME) (η^2^ = 0.887, large) (Table 3).

**Table 3:** Comparison of primary outcomes with intra-group analysis-1

Variable	Sub-dimension	Time	BAT + PR	PR	Time x Group Interaction	Partial η^2^
**LOS**	**RT**	**Before**	0.94 ± 0.07	0.93 ± 0.06	< 0.001*	0.777
		**After**	0.79 ± 0.07	0.92 ± 0.06		
		**p**	< 0.001	0.048		
		**Δ**_**Change**_	-0.14 ± 0.05	-0.01 ± 0.02		
	**MVL**	**Before**	3.40 ± 0.51	3.63 ± 0.67	< 0.001*	0.789
		**After**	4.44 ± 0.45	3.62 ± 0.66		
		**p**	< 0.001	0.741		
		**Δ**_**Change**_	1.03 ± 0.36	-0.01 ± 0.13		
	**EPE**	**Before**	71.63 ± 7.27	72.19 ± 6.51	< 0.001*	0,687
		**After**	85.45 ± 5.71	73.14 ± 6.67		
		**p**	< 0.001	0.079		
		**Δ**_**Change**_	13.81 ± 5.77	0.95 ± 2.35		
	**DC**	**Before**	73.86 ± 5.59	74.00 ± 5.55	0.956	-
		**After**	72.80 ± 5.30	72.90 ± 6.10		
		**p**	0.761	0.874		
		**Δ**_**Change**_	0.13 ± 2.07	0.09 ± 2.71		
	**ME**	**Before**	86.18 ± 8.65	86.23 ± 9.66	< 0.001*	0.887
		**After**	101.36 ± 8.28	85.76 ± 9.54		
		**p**	< 0.001	0.212		
		**Δ**_**Change**_	-15.18 ± 3.63	0.47 ± 1.69		

*BAT* + *PR* Body Awareness Therapy + Pulmonary Rehabilitation Group, *PR* Pulmonary Rehabilitation Group, *LOS* Limit of Stability, *mCTS* Modified Clinical Test of Sensory, *RT* Reaction Time, *MVL* Movement Velocity, *EPE* End-point Excursion, *DC* Directional Control, *ME* Maximum Excursion

η^2^: Partial Eta Squared

^*^Significant difference with *p* < 0.05

When the mCTS scores were examined after the treatment, improvements were observed in static posture skills with eyes open (*p* < 0.001, η^2^ = 0.679, large) and closed (*p* < 0.001, η^2^ = 0.705, large) on firm ground in the BAT + PR group. In contrast, improvements that could not be detected in posture skills were observed with eyes open (*p* = 0.097) and eyes closed (*p* = 0.500) on foam ground in the BAT + PR group. There was a significant interaction effect for the groups by time in Fear of Fall (FoF) (η^2^ = 0.331, large) (Table 4).

**Table 4:** Comparison of primary outcomes with intra-group analysis-2

Variable	Sub-dimension	Time	BAT + PR	PR	Time x Group Interaction	Partial η^2^
**mCTS**	**Firm-EO**	**Before**	0.76 ± 0.17	0.69 ± 0.23	< 0.001*	0.679
		**After**	0.45 ± 0.11	0.71 ± 0.22		
		**p**	< 0.001	0.123		
		**Δ**_**Change**_	0.30 ± 0.15	-0.02 ± 0.06		
	**Firm-EC**	**Before**	0.92 ± 0.18	0.88 ± 0.21	< 0.001*	0.705
		**After**	0.58 ± 0.13	0.88 ± 0.21		
		**p**	< 0.001	0.772		
		**Δ**_**Change**_	0.34 ± 0.14	0.01 ± 0.06		
	**Foam-EO**	**Before**	1.12 ± 0.21	1.02 ± 0.19	0.097	-
		**After**	1.09 ± 0.18	1.03 ± 0.19		
		**p**	0.128	0.480		
		**Δ**_**Change**_	0.03 ± 0.09	-0.01 ± 0.06		
	**Foam-EC**	**Before**	2.36 ± 0.13	2.31 ± 0.21	0.500	-
		**After**	2.35 ± 0.15	2.29 ± 0.20		
		**p**	0.910	0.367		
		**Δ**_**Change**_	0.00 ± 0.07	0.02 ± 0.09		
**FoF**		**Before**	67.04 ± 20.83	64.00 ± 21.65	< 0.001*	0.331
		**After**	51.59 ± 17.80	62.33 ± 20.38		
		**p**	< 0.001	< 0.001		
		**Δ**_**Change**_	15.45 ± 13.15	1.66 ± 4.96		

*BAT* + *PR* Body Awareness Therapy + Pulmonary Rehabilitation Group, *PR* Pulmonary Rehabilitation Group, *mCTS* Modified Clinical Test of Sensory, *FoF* Fear of Falling, *EO* Eyes Open, *EC* Eyes Closed

η^2^: Partial Eta Squared

^*^Significant difference with *p* < 0.05

According to the results of the secondary outcomes, the baseline quadriceps strength score was higher in favor of the PR group (Table 2). However, at the end of the study, the muscle strength of the patients in the BAT + PR group increased more and differed from that of the control group (*p* < 0.001). There was a significant interaction effect for the groups by time in muscle strength (η^2^ = 0.803, large), 6MWT (η^2^ = 0.859, large), and BDS (η^2^ = 0.546, large) (Table 5).

**Table 5:** Comparison of secondary outcomes between groups

Variables	Time	BAT + PR	PR	Time x Group Interaction	Partial η^2^
**Muscle Strenght**	**Before**	17.35 ± 2.01	18.50 ± 1.36	< 0.001*	0.803
	**After**	21.55 ± 2.25	18.49 ± 1.35		
	**p**	< 0.001	0.847		
	**Δ**_**Change**_	4.19 ± 1.47	-0.01 ± 0.22		
**6MWT**	**Before**	426.95 ± 36.89	417.47 ± 23.13	< 0.001*	0.859
	**After**	526.72 ± 38.09	463.14 ± 23.49		
	**p**	< 0.001	< 0.001		
	**Δ**_**Change**_	99.77 ± 12.61	45.66 ± 9.54		
**BDS**	**Before**	4.04 ± 0.89	4.19 ± 1.03	< 0.001*	0.546
	**After**	1.77 ± 0.81	3.52 ± 0.98		
	**p**	< 0.001	< 0.002		
	**Δ**_**Change**_	-2.27 ± 0.63	-0.66 ± 0.85		

*BDS* Borg Dyspnea Scale, *PR* Pulmonary Rehabilitation Group, *ES* Effect Size, *BAT* + *PR* Body Awareness + Pulmonary Rehabilitation Group, *6-MWT* 6-Minutes Walking Test

η^2^: Partial Eta Squared

^*^Significant difference with *p* < 0.05

## Discussion

This study is the first randomized controlled study in which BAT was used as an exercise modality in patients with COPD. The most clinically important finding is that BAT combined with PR is superior to PR alone to improve balance, fear of falling, dyspnea, functional capacity, and muscle strength parameters in patients with COPD.

Much of the available literature suggests that BAT is a creative and enjoyable activity, making rehabilitation more interesting for patients; therefore, it increases the motivation of the patients to continue the program [18]. During exercise, the awareness of movement and the interaction between the mind, body, and behavior can be beneficial in regulating the emotional, mental, social, and behavioral factors that impact health. When we look at the literature, although there are various application protocols of BAT; to allow the patient to experience his/her own body reactions, symptoms, and learned movements, it is recommended to be applied one day a week, 1 h a day [18, 20]. BAT was applied to the BAT + PR group once a week for 1 h in this study.

The role of BAT in the development of balance function has been associated with perturbation training, coordinating movement, strengthening muscles, using different sensory systems, and sudden changes in the center of gravity during exercises performed in different positions [27, 32]. The BAT + PR group showed significant improvements in the eyes open parameters of static balance and in all parameters of dynamic balance. Furthermore, the significant interaction effects between groups according to time were high in reaction time, movement velocity, end-point excursion and maximal excursion.

It was observed that BAT provided significantly more improvement in dynamic balance than in static balance. This may be because BAT exercises include exercises with both eyes open and eyes closed in dynamic positions on different surfaces. In the BAT + PR group, there was no significant difference before and after the treatment in the both mCTS test results performed on a foam surface; there was a significant improvement in the results performed on the firm floor. The Partial eta squared results also proved the large interaction effects over time. Kaya et al. showed that dance movement therapy, and Leung et al. showed that Tai Chi increased balance and functional capacity in patients with COPD [33, 34]. Considering the literature, studies showing the effect of alternative treatment methods on extrapulmonary symptoms in COPD are quite limited, and they appear to be effective when applied with PR.

Many studies have determined that the risk of falls is more common in older patients with COPD [13, 14, 24, 35]. Our findings clearly showed that BAT applied together with PR provided a statistically significant and large-effect improvement in fear of falling compared to the PR group. Beauchamp et al. stated that a standardized 6-week multidisciplinary PR program had no effect on balance confidence in individuals with COPD [36]. On the other hand, Berriet et al. reported that Cognitive Behavioral Therapy given with 4-week PR significantly reduced the fear of falling in COPD patients. Interventions lasted six weeks to 12 months and included muscle strengthening, balance, exercise, stretching, walking, relaxation, and cognitive behavioral therapy [37]. Within the scope of our 8-week program, some similar activities were performed, and at the end of our study; balance gains were achieved and the fear of falling was reduced. In this context, our result is compatible with the literature. We think that this decrease is related to the ease of management of the disease with alternative treatments, the reduction of the stress and emotionally negative components caused by the disease by considering not only the physical but also the psychological dimension.

The number of patients did not allow us to make a comparison between balance and fear of falling levels according to COPD stages. We think that future studies can focus on this issue. Our findings may raise the awareness that pulmonary and extrapulmonary outputs are correlated with each other. Additionally, extrapulmonary functions, as well as pulmonary functions, should be evaluated and included in the treatment plan, and alternative therapies for balance impairments and the fear of falling should be added to rehabilitation programs in COPD.

Patients in both the BAT + PR group and PR groups showed a significant improvement in 6MWT distance improvement. When the distance improvements between the groups were compared, the difference was significantly greater and had a large effect in the BAT + PR group. Since body awareness exercises created an aerobic effort, it is not surprising that the experimental group in our study achieved greater improvement in the 6MWT distance. Improving the 6MWT distance with conventional exercise training in COPD is already known and frequently reported in the literature [38–40].

One of our results showed a significant improvement in the Modified Borg Dyspnea Scale results measured after the 6MWT in both groups. The results of the BAT + PR group were statistically more significant and had a larger effect than those of the PR group. Combining movements with breathing in BAT, learning to perform activities with less energy expenditure with breathing training, and energy conservation techniques may have helped to improve postexercise dyspnea value. Considering the studies examining the effect of alternative treatments applied to patients with chronic obstructive or respiratory pulmonary diseases on dyspnea, practices such as Tai Chi, Yoga, and Mindful Based Therapy integrated into PR significantly improve dyspnea compared to PR alone [34, 41–43].

Failure to evaluate respiratory functions in this study can be considered a limitation. Spirometric evaluations were not included in this study since the study was carried out during the pandemic period and the risk of transmission.

## Conclusion

To our knowledge, this is the first study to evaluate the effect of BAT on balance and fear of falling in patients with COPD. The results of this study show that BAT, in addition to traditional PR, was safe and had significantly clear benefits to increase balance and reduce fear of falling in patients with COPD. We suggest that BAT, an alternative intervention with various exercises appealing to individuals of all ages, should be presented as an alternative option for pulmonary rehabilitation program content for those who find it more enjoyable or motivating than other aerobic exercises and meditative exercise types.

### Supplementary Information

**Supplementary Materials 1.**

## Data Availability

The datasets used and/or analyzed during the current study are available from the corresponding author (SK) upon reasonable request. Because it contains personal information about the patients.

## Abbreviations

COPD: Chronic Obstructive Pulmonary Disease
GOLD: Global Initiative for Obstructive Lung Diseases
PR: Pulmonary rehabilitation
BAT: Body Awareness Therapy
mCTS: Modified Clinical Test of Sensory
LOS: Limits of Stability Test
FES-I: Fall Efficiency Scale International
BDS: Borg Dyspnea Scale
6MWT: 6 Minute walking test
FoF: Fear of Fall
DC: Directional Control
RT: Reaction Time
CAT: COPD Assessment Test
mMRC: Modified Medical Research Council
