# Supplementary material for: Effects of body awareness therapy on balance and fear of falling in patients with chronic obstructive pulmonary disease: a randomized controlled trial
Source: Biopsychosoc Med. 2024 Feb 26;18:6. doi: 10.1186/s13030-024-00303-x (PMC10895779; doi:10.1186/s13030-024-00303-x)
Supplement: Supplementary file 1 — Supplementary Materials 1. [file 13030_2024_303_MOESM1_ESM.docx]

**Supplement 1**

**Body Awareness Therapy Treatment Program**

| **Week** | **Program** |
| --- | --- |
| First week | Free breathing, body scan meditation, comforting touch, walking backward, massage, and mental awareness. |
| Second week | Diaphragmatic breathing, body scan meditation, comforting touch, grounding exercises, progressive muscle relaxation, massage, and mental awareness. |
| Third week | Diaphragmatic breathing, body scan meditation, bilateral movement, postural stability training, progressive muscle relaxation, massage, and mental awareness. |
| Fourth week | Diaphragmatic breathing, body scan meditation, bilateral movement, balance exercises, movement meditation, massage, and psychic sleep. |
| Fifth week | Diaphragmatic and thoracic breathing, body scan meditation, progressive bilateral movement, balance exercises, coordination training with rhythm, massage, psychic sleep. |
| Sixth week | Diaphragmatic and thoracic breathing, body scan meditation, clinical somatics exercises, Postural stability training, massage, focus on movement quality, “how the movements are performed in relation to space, time, and energy. |
| Seventh week | Diaphragmatic and thoracic breathing, body scan meditation, proprioception exercises, static balance training, massage, focus on movement quality, “how the movements are performed in relation to space, time, and energy. |
| Eighth week | Diaphragmatic and thoracic breathing, body scan meditation, proprioception exercises, dynamic balance training, massage, focus on movement quality, “how the movements are performed in relation to space, time, and energy. |
